# Identifying the Phytotoxicity of Biosynthesized Metal Oxide Nanoparticles and Their Impact on Antioxidative Enzymatic Activity in Maize Under Drought Stress

**DOI:** 10.3390/plants14071075

**Published:** 2025-04-01

**Authors:** Hafiz Muhammad Rizwan, Usman Shafqat, Aneeza Ishfaq, Fatima Batool, Faisal Mahmood, Qitao Su, Nimra Yaseen, Tehziba Raza, Faizah Amer Altihani

**Affiliations:** 1 Department of Environmental Sciences, Government College University Faisalabad, Faisalabad 38000, Pakistan; hafizmrizwan0042@gmail.com (H.M.R.); usmanshafqat@gcuf.edu.pk (U.S.); aneeza.201703601@gcuf.edu.pk (A.I.); fatima.201602599@gcuf.edu.pk (F.B.); nimrayaseen266@gmail.com (N.Y.);; 2 School of Life Sciences, Key Laboratory of Jiangxi Province for Biological Invasion and Biosecurity, Jinggangshan University, Ji’an 343009, China; 3 Department of Biology, College of Science, King Khalid University, Abha 61413, Saudi Arabia; ftehany@kku.edu.sa

**Keywords:** cereal crop, *Conocarpus erectus* L., abiotic stress, sustainable agriculture, metal oxide nanoparticles

## Abstract

Maize (*Zea mays* L.), an important crop used for animal feed and human consumption, is currently threatened by water shortage. Recently, the usage of nanomaterials has attracted worldwide attention due to their applications in various fields. This research aimed to evaluate the comparative efficacy of different metal oxide nanoparticles for mitigating drought stress in maize. Iron oxide, manganese oxide, and copper nanoparticles were biosynthesized from the leaf extract of *Conocarpus erectus* L. and characterized via UV-Vis, XRD, FTIR, and SEM. The synthesized nanomaterials were initially optimized at different concentrations (0, 25, 50, 75, and 100 ppm). The optimized doses of each nanoparticle were then applied to maize plants under different drought stress levels (50% FC, 75% FC, and 100% FC). Compared to the control, the application of nanomaterials significantly improved the growth parameters of the maize by 30% at 50% FC, 27% at 75% FC, and 26% at 100% FC. The chlorophyll content also improved significantly at different levels of drought stress by 35%, 32%, and 29% as compared to the control, respectively. The antioxidants (CAT, POD, SOD, and APX) also improved significantly at different levels of drought by 37%, 34%, and 31%, as compared to control, respectively. Moreover, the use of nanoparticles resulted in a significant decrease in cellular oxidative stress (MDA, H_2_O_2_) parameters by 23% at 50%FC, 26% at 75% FC, and 27% at 100% FC. Biosynthesized FeO NPs, MnO NPs, and Cu NPs have demonstrated significant potential in mitigating drought stress in maize, suggesting a promising approach to enhance crop performance under water-limited conditions. Further research is recommended to explore the long-term impacts and practical applications of these findings in sustainable agriculture.

## 1. Introduction

Agriculture plays a crucial role in Pakistan’s gross domestic product (GDP). It also supports the country’s economic growth while addressing global food challenges. Maize is a vital crop due to its nutritional value, which provides essential carbohydrates, vitamins, and minerals [1]. In addition to human consumption, maize is also used in livestock feed production. It is also used as feed and food for animal nourishment [2]. Globally, maize is an important cereal with an annual production exceeding 1.2 billion metric tons, contributing to food security and the economy [3]. It is highly sensitive to drought, with yield losses ranging from 20 to 50% under severe drought stress. Maize requires approximately 500–800 mm of water per season, making it vulnerable to water scarcity [4]. Maize is a C4 plant that requires a significant amount of water for irrigation. Drought stress is one of the yield-limiting factors in maize crops [5]. Over the past few decades, drought has become one of the severe stresses caused by climate change [6]. It negatively impacts physical growth parameters such as leaf size, stem height, and overall biomass by disrupting water uptake, nutrient absorption, and metabolic functions [7]. Water scarcity also induces oxidative stress that leads to lipid membrane degradation [8]. Lipid oxidation consequently reduces membrane permeability, ultimately decreasing plant metabolism and yield [9].

Reducing drought stress is important for increasing crop yield. Conventionally, drought stress is addressed through methods such as drip irrigation, sprinkler systems, and osmo-protectants which include glutathione, glycine betaine, proline, and trehalose [10]. Due to their high initial cost, installation expenses for conventional systems pose a serious challenge. Thus, there should be new, cost effective, and sustainable methods for the alleviation of drought stress [7]. Recently, nanotechnology has been considered a potential technology in agriculture for numerous applications. NPs have a large surface area-to-volume ratio, increasing their bioavailability in agriculture systems. They can be applied in agriculture as biofertilizers, biopesticides, anti-stresses, and antimicrobial agents [11]. NPs can be synthesized via physical, chemical, and biological methods. The only difference between all these methods is the selection of a reducing agent. In the case of synthesis, there are two mandatory things: one is precursor salt, and the other is a reducing agent [12]. In biological synthesis, reducing agents are extracted from plants, bacteria, or other metabolites present in the biological system [13]. The green synthesis of nanoparticles from plants is a more convenient method because of their easy availability in large quantities [14].

Iron (Fe), manganese (Mn), and copper (Cu) nanoparticles have emerged as vital solutions in agriculture for increasing the germination rate and plant growth under abiotic and biotic stress conditions [15,16]. Ishfaq et al. [6] synthesized biogenic zinc oxide nanoparticles to alleviate drought stress. The results revealed a 37% reduction in drought stress when these nanoparticles were used to improve pea growth. According to the work of Semenova et al. [17], Fe NPs and Mn NPs enhance plant production through improved nutrient absorption. In this study, FeO, MnO, and Cu NPs were synthesized through biological methods because of their multipurpose use in various fields of agriculture, medicine, and environmental conservation. Iron plays a vital role in physiological activities such as respiration and photosynthesis, and its deficiency affects plant growth [11,18]. FeO NPs also supply iron directly to particular plant tissues that require iron for their activity [19]. Mn NPs are utilized in industries-specific batteries, redox-catalyst applications, biomedical imaging, and as antioxidants to increase the germination of seedlings and plant stress tolerance [20]. Cu NPs can increase water potential through their impact on the cell membranes of plants to mitigate drought stress. It is also observed that Cu NPs can reduce the deterioration of cell membranes, ion transport, and nutrient uptake, thus enhancing plant’s growth under drought stress [21]. Moreover, Cu NPs help to minimize water loss through stomata by increasing the concentration of ABA and stomata closure, preserving plants from oxidative stress and increasing the level of ROS [22]. Furthermore, Cu NPs affect the plant stress defense system related to drought stress and therefore increase anthocyanin, chlorophyll, and carotenoid contents, as well as plant biomass and grain yield under drought stress. Cu NPs also regulate the metabolic processes in plants, such as photosynthesis and enzyme activation [23]. In previous studies, many scientists have used metal oxide nanoparticles to mitigate drought stress in different crops. Kandhol et al. [24] reported that the application of metal oxide nanoparticles can significantly increase root length and weight, and chlorophyll content, modulate gene expression, minimize the loss of water via stomatal closure, and improve crop yield under drought stress. El-Saadony et al. [25] reported that drought stress decreased the vessel diameter, stem length, and width of sunflowers. They found that foliar application of Zn NPs mitigated drought stress and significantly improved the epidermis thickness, vascular bundle size and width, and vessel diameter of sunflowers.

The drawback of using nanoparticles is their phytotoxic threshold [26]. The thresholds of different nanoparticles are different for different crops [27]. The adaptive mechanism of maize under drought conditions is one of the major concerns of agricultural researchers due to the increasing incidence of water scarcity globally. To make nanoparticles useful and safe for plant applications, they need to be optimized first. Higher concentrations of nanoparticles can be toxic to the plants, potentially causing damage to their cells, tissues, and overall health of the plants. Optimized doses of nanoparticles can improve nutrient uptake, reduce pest and disease attacks [28], and increase environmentally friendly farming methods. In previous studies, researchers have used different nanoparticles to mitigate drought stress in different crops. We hypothesized that the application of optimized iron oxide nanoparticles (FeO NPs), manganese oxide nanoparticles (MnO NPs), and copper nanoparticles (Cu NPs) could help in minimizing the drought stress in the maize. Therefore, this study was conducted with the following objectives: (i) to determine the impacts of optimized concentration of FeO, MnO, and Cu NPs on morphological growth, chlorophyll synthesis, oxidative stress markers, and antioxidant activities of maize grown under drought stress, (ii) to compare the efficacy of biosynthesized NPs in determining the threshold needed to mitigate their negative impacts.

## 2. Results

### 2.1. Biosynthesis and Characterization of Nanoparticles

**Figure 1:**
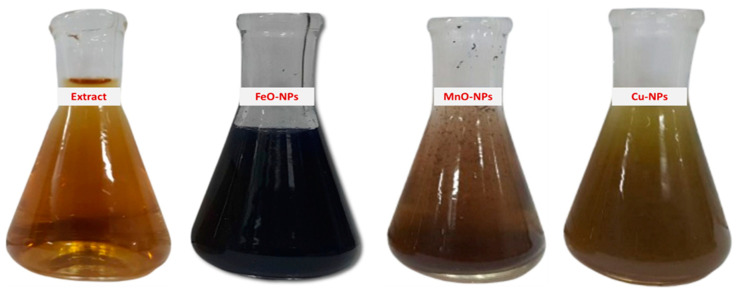
Color indication of biosynthesized iron oxide, manganese oxide, and copper nanoparticles.

**Figure 2:**
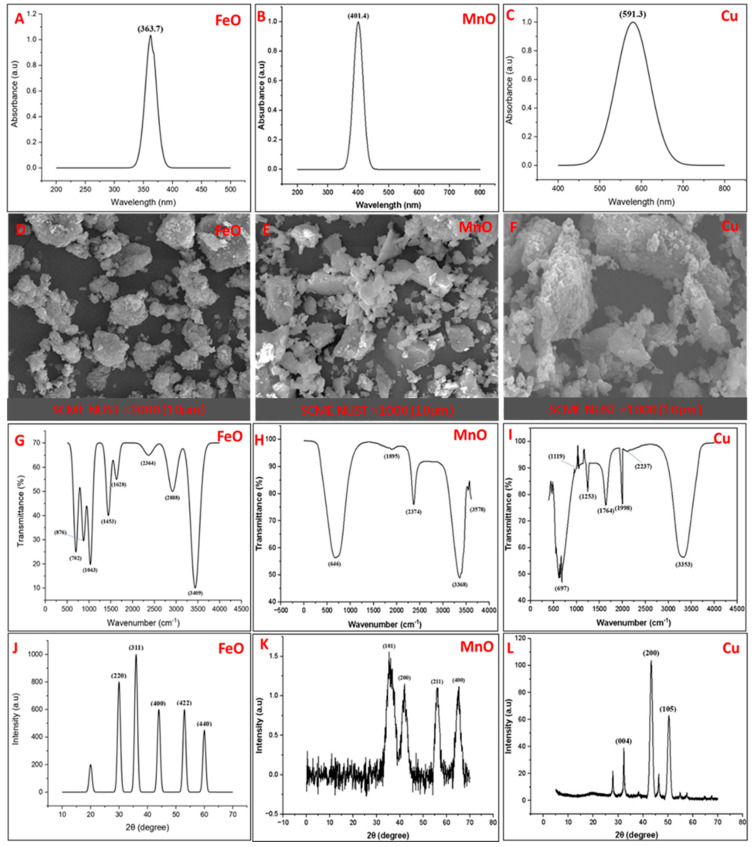
Characterization of iron oxide nanoparticles via (**A**) UV-visible spectroscopy, (**D**) scanning electron microscopy, (**G**) Fourier transform infrared (FTIR) spectroscopy, and (**J**) X-ray diffraction (XRD); manganese oxide nanoparticles via (**B**) UV-visible spectroscopy, (**E**) scanning electron microscopy, (**H**) FTIR spectroscopy, and (**K**) X-ray diffraction; and copper nanoparticles via (**C**) UV-visible spectroscopy, (**F**) scanning electron microscopy, (**I**) FTIR spectroscopy, and (**L**) X-ray diffraction.

The synthesis of FeO, MnO, and Cu NPs was initially confirmed by the color change: the FeO color changed from yellow to black, the MnO changed from yellow to brownish, and the Cu color changed from yellow to greenish (Figure 1). UV-Vis spectrum further confirmed the synthesis of nanoparticles with distinct absorption peaks at 363 nm (FeO), 401 nm (MnO), and 590 nm (CuO) (Figure 2A–C). These peaks resemble the characteristic electronic transitions of metal oxide nanoparticles and align with previously reported values [6,26]. The blueshift observed in the FeO and MnO NPs suggests a potential reduction in particle size due to quantum confinement effects. SEM analysis revealed that the FeO NPs exhibited irregular, aggregated structures with a rough surface texture. The MnO NPs displayed a highly aggregated morphology with angular and flake-like shapes. The Cu NPs had an irregular shape with significant agglomeration and clustered structures (Figure 2D–F). The FTIR analysis of FeO NPs revealed peaks at 3409 cm^−1^ (-OH stretching), 2888 cm^−1^ (C-H stretching), 1628 cm^−1^ (H-O-H bending), 1453 cm^−1^ (C-H deformation), 1043 cm^−1^ (C-O stretching), and 702 cm^−1^ (Fe-O stretching), (Figure 2G). When analyzing MnO NPs (Figure 2H), peaks were observed at 646 cm^−1^ (Mn-O stretching), 3368 cm^−1^ (O-H stretching from alcohol), 1895 cm^−1^, and 2374 cm^−1^, indicating metal–ligand interactions and possible organic capping agents. FTIR analysis of the Cu NPs (Figure 2I) showed a peak at 3353, which was assigned to the OH group. The peak at 1764 cm^−1^ was due to C-C stretching. The peak at 697 cm^−1^ might be due to the formation of Cu or CuO NPs. These spectral features indicate the presence of organic capping agents that may contribute to nanoparticle stability and dispersion. Additionally, the X-ray diffraction analysis revealed peaks at 220 (30°), 311 (36°), 400 (44°), 422 (53°), and 440 (60°), indicating the crystalline nature of FeO NPs (Figure 2J). The XRD peaks at 101 (36°), 200 (42°), 211 (56°), and 511 (66°) confirmed the crystalline nature of MnO NPs (Figure 2K). For copper, the peaks at 111, 200, and 220 were assigned to the 44.5°, 51.5°, and 74.75°, confirming the crystallinity of the particles. The crystallite sizes of the nanoparticles were determined via the Scherrer equation, yielding 25 nm for FeO NPs, 18 nm for MnO NPs, and 30 nm for CuO NPs (Figure 2L).

### 2.2. Optimization of Nanoparticles

#### 2.2.1. Morphological Growth

**Figure 3:**
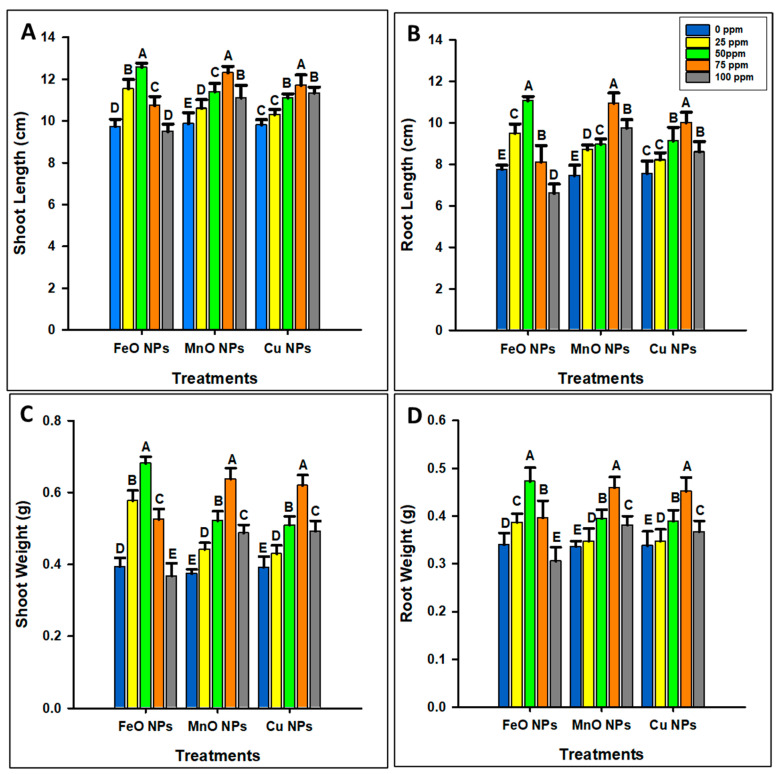
Optimization of different concentrations of iron oxide, manganese oxide, and copper nanoparticles by estimating (**A**) shoot length, (**B**) root length, (**C**) shoot weight, and (**D**) root weight of *Zea mays* L. crop. The data are the mean of three replications (n = 3) with ± standard deviations. Different letters above the bars indicate significance differences (*p* ≤ 0.05) among means according to Fisher’s LSD.

The application of FeO NPs significantly increased the shoot length (SL), root length (RL), shoot weight (SW), and root weight (RW) at 50 ppm concentration and then decreased them, as shown in Figure 3A–D. The maximum SL (12.6 cm), RL (11 cm), SW (0.6810 g), and RW (0.4723 g) were observed at 50 ppm. The minimum SL (9.5 cm), RL (6.6 cm), SW (0.3670 g), and RW (0.3053 g) were observed at 100 ppm concentration. However, the application of MnO NPs improved the physical growth parameters, as the maximum SL (12.3 cm), RL (10.9 cm), SW (0.6370 g), and RW (0.4570 g) were observed at 75 ppm and minimum (9.9 cm, 7.5 cm, 0.4870 g, and 0.3810 g) at 0 ppm concentration. Similarly, the application of 75 ppm Cu NPs significantly improved the morphological parameters, which subsequently decreased. The maximum SL (11.7 cm), RL (10 cm), SW (0.6193 g), and RW (0.4517 g) were observed at 75 ppm concentration, and the minimum (9.8 cm, 7.6 cm, 0.4917 g, and 0.3670 g) were observed at 0 ppm. Overall, FeO NPs demonstrated a phytotoxic threshold of 50 ppm, whereas MnO NPs and Cu NPs showed a higher threshold of 75 ppm.

#### 2.2.2. Chlorophyll Contents

**Figure 4:**
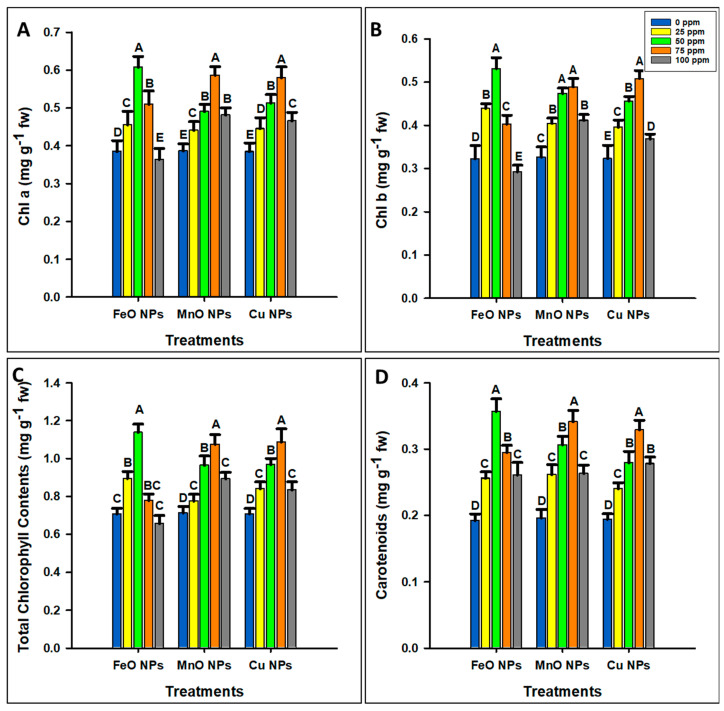
Optimization of different concentrations of iron oxide, manganese oxide, and copper nanoparticles by estimating (**A**) chlorophyll a, (**B**) chlorophyll b, (**C**) total chlorophyll content, and (**D**) carotenoids in *Zea mays* L. crop. The data are the mean of three replications (n = 3) with ± standard deviations. Different letters above the bars indicate significant differences (*p* ≤ 0.05) among means according to Fisher’s LSD.

The application of FeO NPs significantly increased the chlorophyll and carotenoid contents at 50 ppm concentration and then decreased them, as shown in Figure 4A–D. The maximum chlorophyll a (0.6073 mg g^−1^ fw), b (0.5307 mg g^−1^ fw), total chlorophyll content (1.1380 mg g^−1^ fw), and carotenoids (0.3570 mg g^−1^ fw) were observed at 50 ppm concentration, whereas the minimum chlorophyll a (0.3637 mg g^−1^ fw), b (0.2920 mg g^−1^ fw), and total chlorophyll content (0.6557 mg g^−1^ fw) were observed at 100 ppm concentration and for carotenoids (0.1920 mg g^−1^ fw) at 0 ppm concentration. Moreover, the application of MnO NPs improved the chlorophyll a, b, total chlorophyll, and carotenoids at 75 ppm concentration, followed by 50 ppm concentration, and the minimum (0.3860, 0.3263, 0.7123, 0.1960 mg g^−1^ fw) was observed at 0 ppm concentration. Similarly, the application of Cu NPs significantly improved the photosynthetic pigment content at 75 ppm concentration and then decreased. The maximum chlorophyll a (0.5793 mg g^−1^ fw), b (0.5080 mg g^−1^ fw), total chlorophyll (0.1.0873 mg g^−1^ fw), and carotenoids (0.3287 mg g^−1^ fw) were observed at 75 ppm concentration. Minimum chlorophyll a (0.3843 mg g^−1^ fw), b (0.3230 mg g^−1^ fw), total chlorophyll (0.7073 mg g^−1^ fw), and carotenoids (0.1940 mg g^−1^ fw) contents were observed at 0 ppm. Overall, FeO NPs demonstrated a phytotoxic threshold of 50 ppm, whereas MnO NPs and Cu NPs presented a higher threshold of 75 ppm.

#### 2.2.3. Antioxidant

**Figure 5:**
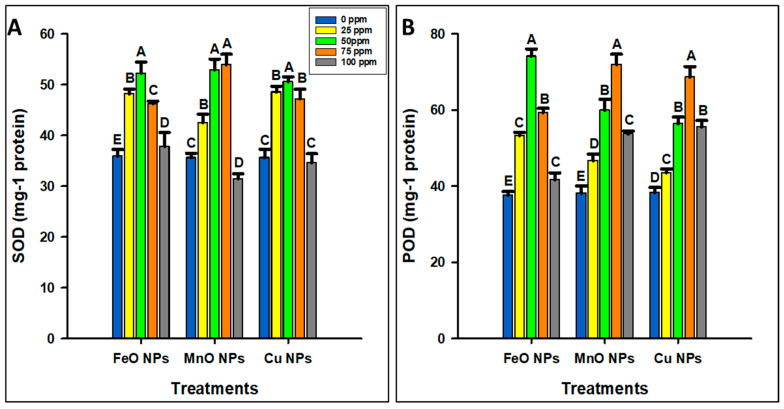
Optimization of different concentrations of iron oxide, manganese oxide, and copper nanoparticles by estimating (**A**) superoxide dismutase (SOD), and (**B**) peroxidase (POD) in *Zea mays* L. crop. The data are the mean of three replications (n = 3) with ± standard deviations. Different letters above the bars indicate significant differences (*p* ≤ 0.05) among means according to Fisher’s LSD.

The application of FeO NPs significantly increased the SOD and POD activities at 50 ppm concentration but decreased them at other concentrations (Figure 5). The maximum concentrations of SOD (52.2 mg^−1^) and POD (74.14 mg^−1^) were observed at 50 ppm, and the minimum concentration (37.82 and 37.70 mg^−1^) were observed at 0 ppm. Additionally, the application of MnO NPs improved the SOD (53.94 mg^−1^ protein) and POD (71.98 mg^−1^ protein) at 75 ppm concentration, followed by 50 ppm. The minimum SOD activity (31.42 mg^−1^ protein) was observed at 100 ppm, and the minimum POD activity (38.16 mg^−1^ protein) was observed at 0 ppm concentration. However, the application of Cu NPs significantly improved the SOD (50.57 mg^−1^) and POD (68.66 mg^−1^) contents at 75 ppm concentration, which then decreased. The minimum SOD (34.61 mg^−1^ protein) was observed at 100 ppm concentration, and the POD activity (38.36 mg^−1^ protein) was lowest at 0 ppm concentration. Overall, FeO NPs demonstrated a phytotoxic threshold of 50 ppm, whereas MnO NPs and Cu NPs presented a higher threshold of 75 ppm.

#### 2.2.4. Oxidative Stress

**Figure 6:**
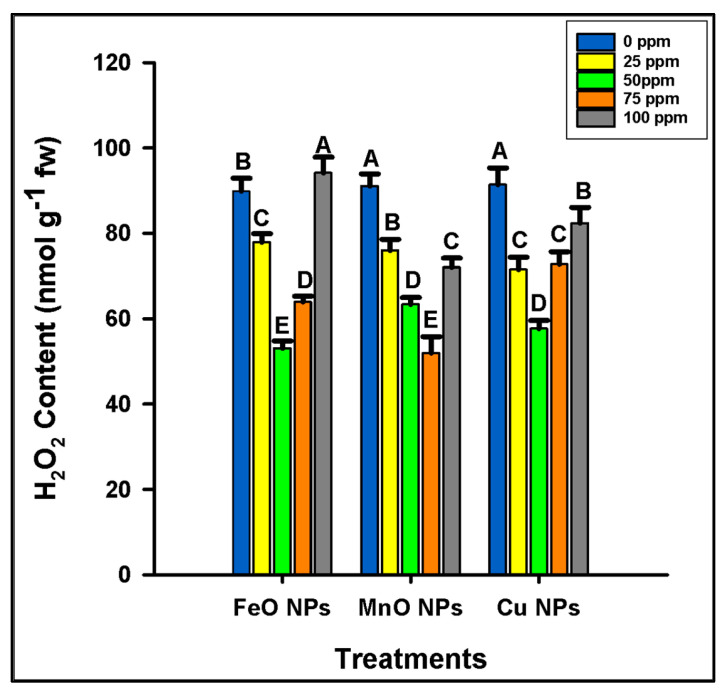
Optimization of different concentrations of iron oxide, manganese oxide, and copper nanoparticles by estimating hydrogen peroxide (H_2_O_2_) in *Zea mays* L. crop. The data are the mean of three replications (n = 3) with ± standard deviations. Different letters above bars indicate the significant differences (*p* ≤ 0.05) among means according to Fisher’s LSD.

The application of FeO NPs significantly decreased hydrogen peroxide (H_2_O_2_) stress at 50 ppm concentration. The maximum H_2_O_2_ stress (94.12 nmol g^−1^ fw) was observed at 100 ppm concentration (Figure 6). The minimum H_2_O_2_ stress was observed (53.03 nmol g^−1^ fw) at 50 ppm concentration. In the case of MnO NPs, the maximum H_2_O_2_ stress was observed (91.33 nmol g^−1^ fw) at 0 ppm concentration, and the minimum H_2_O_2_ stress (57.64 nmol g^−1^ fw) was observed at 50 ppm concentration. Similarly, Cu NPs application significantly reduced the H_2_O_2_ stress at 75 ppm concentration. The maximum H_2_O_2_ stress was observed (91.33 nmol g^−1^ fw) at 0 ppm concentration and minimum (57.64 nmol g^−1^ fw) at 50 ppm concentration. The oxidative stress parameter confirmed the phytotoxic threshold of FeO NPs at 50 ppm, whereas the phytotoxic threshold of MnO and Cu NPs was 75 ppm (Figure 6).

### 2.3. Experiment Results

#### 2.3.1. Morphological Parameter

**Figure 7:**
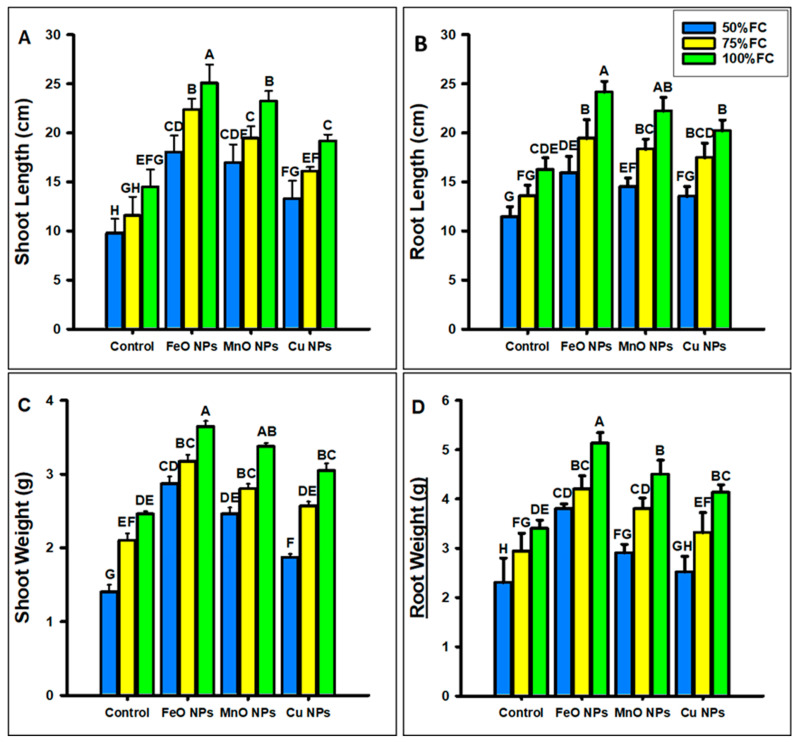
Effect of foliar application of different concentrations of iron oxide, manganese oxide, and copper nanoparticles against drought stress on (**A**) shoot length, (**B**) root length, (**C**) shoot weight, and (**D**) root weight in *Zea mays* L. crop. The data are the mean of three replications (n = 3) with ± standard deviations. Different letters above the bars indicate the significant differences (*p* ≤ 0.05) among means according to Fisher’s LSD.

The foliar application of FeO, MnO, and Cu NPs significantly improved the physical growth parameters of maize under different levels of drought stress (50%, 75%, and 100% FC), as shown in Figure 7A–D. At 50% FC, FeO NPs increased the growth parameters (SL, RL, FSW, and FRW) by 45%, 28%, 51%, and 39%; MnO NPs by 42%, 21%, 43%, and 20%; and Cu NPs increase them by 26%, 15%, 25%, and 8%, respectively, under drought stress. At 75% FC, the addition of FeO NPs increased the growth parameters (SL, RL, FSW, and FRW) by 48%, 30%, 33%, and 30%; the addition of MnO NPs by 40%, 25%, 25%, and 22% and the addition of Cu NPs increased them by 28%, 22%, 18%, and 11%, respectively. At 100% FC, FeO NPs increased the growth parameters (SL, RL, FSW, and FRW) by 42%, 32%, 32%, and 33%; MnO NPs by 37%, 26%, 27%, and 24% and Cu NPs increased them by 24%, 19%, 19%, and 17%, respectively, at different levels of drought stress. The FeO NPs showed the best results, followed by MnO and Cu nanoparticles.

#### 2.3.2. Photosynthetic Pigments and SPAD Values

**Figure 8:**
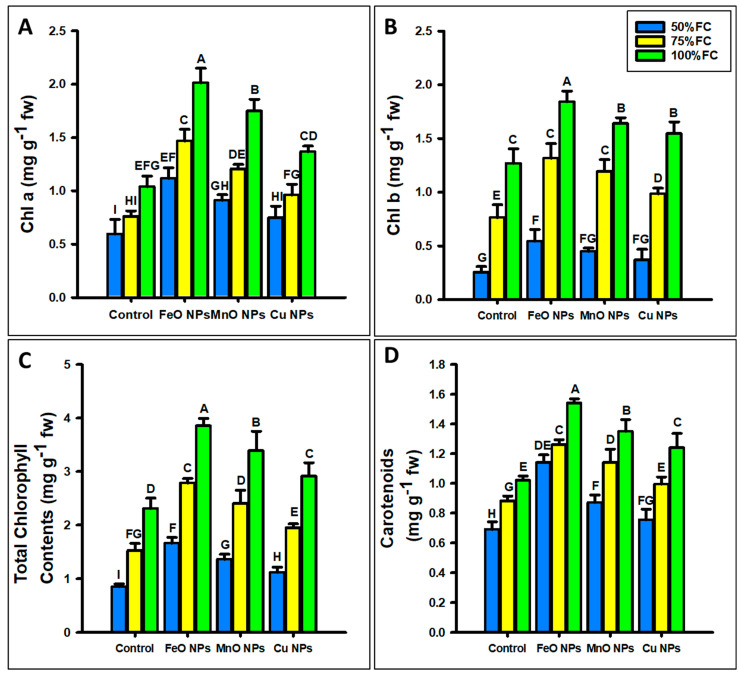
Effect of foliar application of different concentrations of iron oxide, manganese oxide, and copper nanoparticles against drought stress on (**A**) chlorophyll a, (**B**) chlorophyll b, (**C**) total chlorophyll content, and (**D**) carotenoids in *Zea mays* L. crop. The data are the mean of three replications (n = 3) with ± standard deviations. Different letters above the bars indicate the significant differences (*p* ≤ 0.05) among means according to Fisher’s LSD.

Foliar application of FeO, MnO, and Cu NPs significantly improved the photosynthetic content (Figure 8) of maize under different levels of drought stress (50%, 75%, and 100% FC). At 50% FC, FeO NPs increased the photosynthetic content (chl a, b, total chlorophyll, and carotenoids) by 46%, 53%, 48%, and 44%; MnO NPs by 34%, 43%, 37% and 31%, and Cu NPs by 20%, 31%, 23% and 22% at 50%, respectively, under drought stress. At 75% FC, FeO NPs increased the chlorophyll content parameters (chl a, b, total chlorophyll, and carotenoids) by 48%, 42%, 45%, and 42%; MnO NPs by 37%, 36%, 46%, and 31%; and Cu NPs by 21%, 22%, 21%, and 23%, respectively. At 100% FC, the FeO NPs increased the chlorophyll content parameters (chl a, b, total chlorophyll, and carotenoids) by 48%, 31%, 40%, and 40%; the MnO NPs increased the chlorophyll content by 40%, 22%, 31%, and 30%; and the Cu NPs increased the chlorophyll contents by 23%, 17%, 20%, and 21%, respectively, at different levels of drought stress. FeO NPs showed the best results, followed by MnO and Cu nanoparticles.

#### 2.3.3. Antioxidant Parameters

**Figure 9:**
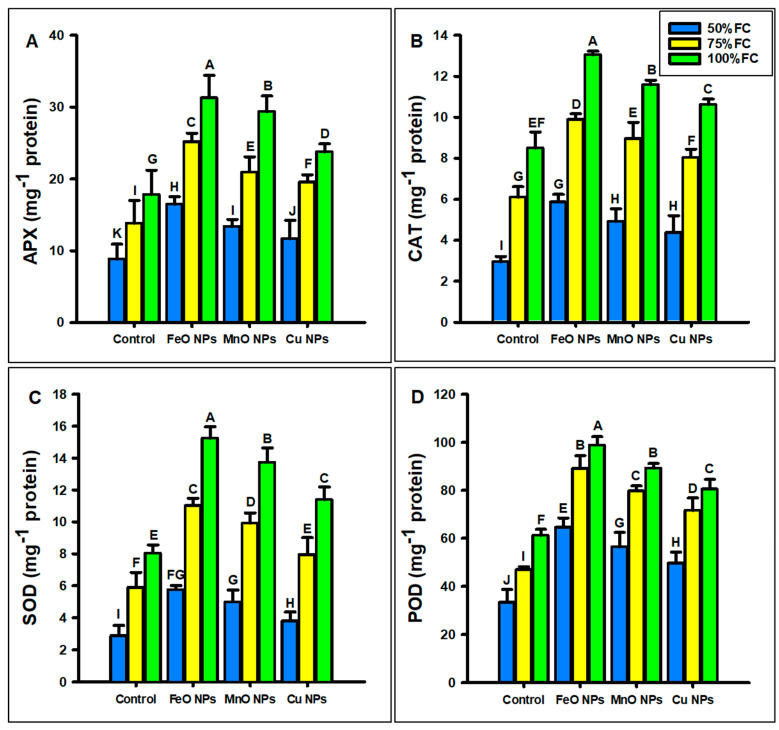
Effect of foliar application of different concentrations of iron oxide, manganese oxide, and copper nanoparticles against drought stress on (**A**) ascorbate peroxidase (APX), (**B**) catalase (CAT), (**C**) superoxide dismutase (SOD), and (**D**) peroxidase (POD) on *Zea mays* L. crop. The data are the mean of three replications (n = 3) with ± standard deviations. Different letters above bars indicate the significant differences (*p* ≤ 0.05) among means according to Fisher’s LSD.

The foliar application of FeO, MnO, and Cu NPs significantly improved the antioxidant parameters of maize under different levels of drought stress (50%, 75%, and 100% FC), as shown in Figure 9A–D. The experiment was conducted with three biological replicates, and the data were analyzed using ANOVA, followed by standard deviation calculations, to ensure statistical reliability. At 50% FC, FeO NPs increased the antioxidants (APX, CAT, SOD, and POD) by 46%, 49%, 49%, and 48%; MnO NPs by 34%, 40%, 41%, and 40%; and Cu NPs by 24%, 32%, 24%, and 32% at 50%, respectively, under drought stress. At 75% FC, FeO NPs increased the antioxidants parameters (APX, CAT, SOD, and POD) by 45%, 38%, 46%, and 47%; MnO NPs by 34%, 31%, 40%, and 41%; and Cu NPs by 29%, 24%, 25%, and 34%, respectively. At 100% FC, FeO NPs increased the antioxidants parameters (APX, CAT, SOD, and POD) by 43%, 34%, 47%, and 38%; MnO NPs by 39%, 26%, 41%, and 31%; and Cu NPs by 25%, 19%, 29%, and 23%, at different levels of drought stress. The FeO NPs showed the best results, followed by MnO and Cu nanoparticles.

#### 2.3.4. Oxidative Stress Parameters

**Figure 10:**
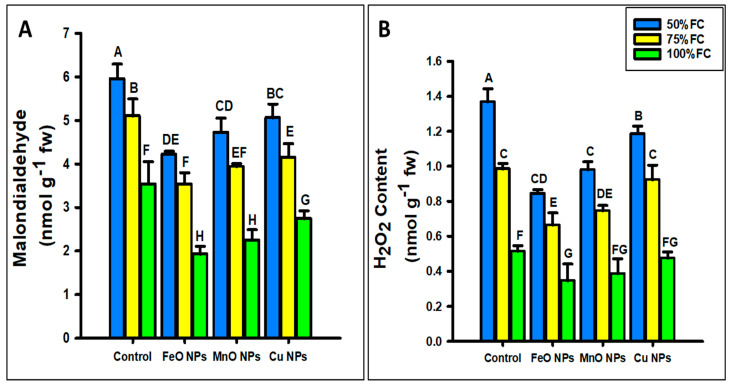
Effect of foliar application of different concentrations of iron oxide, manganese oxide, and copper nanoparticles against drought stress on (**A**) malondialdehyde (MDA) and (**B**) hydrogen peroxide (H_2_O_2_) in *Zea mays* L. crop. The data are the mean of three replications (n = 3) with ± standard deviations. Different letters above the bars indicate the significant differences (*p* ≤ 0.05) among means according to Fisher’s LSD.

The foliar application of FeO, MnO, and Cu NPs significantly decreased the oxidative stress parameters in maize under different levels of drought stress (50%, 75%, and 100% FC), as shown in Figure 10. The experiment was conducted with three replicates, and the data were analyzed via ANOVA, followed by standard deviation calculations, to ensure statistical reliability. At 50% FC, FeO NPs increased the oxidative parameters (MDA and H_2_O_2_) by 29% and 38%; MnO NPs by 20% and 28%; and Cu NPs by 15% and 13% at 50%, respectively, under drought stress. At 75% FC, FeO NPs decreased the oxidant parameters (MDA and H_2_O_2_) by 30% and 32%; MnO NPs by 22% and 24%; and Cu NPs by 18% and 6%, at different levels of drought stress. At 100% FC, FeO NPs decreased the MDA and H_2_O_2_ by 45% and 32%; MnO NPs by 36% and 24%; and Cu NPs by 22% and 7%, respectively, at different levels of drought stress. The FeO NPs showed the lowest results, followed by MnO and Cu NPs.

## 3. Discussion

Maize is an important crop that is significantly affected by abiotic stresses, especially drought. This study was designed to explore the potential of biosynthesized iron oxide, manganese oxide, and copper nanoparticles as an innovative solution to drought. Initially, the nanoparticles were synthesized using *Conocarpus erectus*. Shafqat et al. [26] explored the potential of *Conocarpus erectus* for the synthesis of different nanomaterials. The FeO, MnO, and Cu NPs showed absorption peaks at 363, 401, and 590 nm. Murgueitio-Herrera et al. [29] reported the absorption of FeO-NPs at 360 nm. In the case of MnO-NPs, Ishfaq et al. [20] observed an absorption peak between 300 and 500 nm. Moreover, Batool et al. and Yin et al. [30,31] reported the absorption peak of Cu-NPs at 325 nm. Biosynthesis conditions and capping agents may have an impact on the particle’s size, shape, and surface chemistry composition. SEM analysis revealed an irregular spherical shape of nanomaterials with agglomeration and a flake-like appearance. The agglomeration of particles might be due to high surface area and a lack of stabilizing agents. Similarly, Shafqat et al. [26] reported the flaky, cylindrical shape of FeO NPs with considerable aggregations. Ishfaq et al. [20] revealed that MnO-NPs were 21–30 nm in size with an agglomeration between large particles. However, flake-like structures of Cu-NPs were observed by Shafqat et al. [26]. FTIR analysis revealed the attachment of various functional groups on the surface of the biosynthesized nanomaterials. Elumalai et al. and Osadebe et al. [32,33] reported the attachment of -OH, H-C, and H-C=O groups to the surface of FeO-NPs. Saod et al. [34] observed peaks at 613 and 570 cm^−1^ that were due to the stretching of Mn-O and O-Mn-O bonds. Nieto-Maldonado et al. [35] synthesized Cu NPs from plant extracts and observed numerous absorption peaks, such as 3271, 2931, 1606, and 1029 cm^−1^. The O-H and C-H bond peaks were detected at 3271 and 2931 cm^−1^, respectively. However, the major peaks at 1606 and 1029 cm^−1^ originated from C-O bond. Finally, the XRD analysis revealed the crystalline nature of FeO, MnO, and Cu NPs. The same results were reported by Rama et al. [36]. They observed diffraction peaks of FeO NPs at 220, 311, 400, 422, and 440°, and several other peaks were also observed. Ishfaq et al. [20] observed diffraction peaks of MnO NPs which were synthesized from *Bacillus flexus* at 101, 201, 210, 211, and 511.

**Figure 11:**
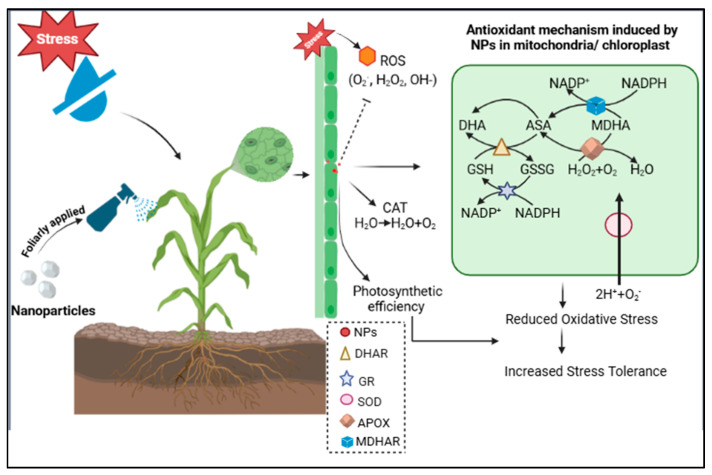
Schematic representation of the antioxidant mechanism induced by nanoparticles in maize under drought stress. This figure was created with BioRender.com.

The adaptive mechanisms of maize under drought conditions have been the focus of agricultural research because of the increasing issue of water scarcity globally. In this context, the optimization of nanoparticles is essential to make them effective and safe in increasing plant resilience and productivity. The non-optimized dosage of nanoparticles can damage the plants, as they may have toxic effects on the cells and tissues of plants, eventually compromising overall growth and health [37]. Additionally, optimized nanoparticles improve nutrient uptake, reduce pests and diseases, and increase the environmentally friendly farming methods [38]. FeO, MnO, and Cu NPs significantly increased the physical growth parameters (shoot length, root length, shoot weight, and root weight) of maize at optimal concentrations, with FeO NPs showing maximum growth at 50 ppm, and MnO and Cu NPs at 75 ppm. The application of NPs improved the growth parameters as they are vital for enzyme activation, chlorophyll synthesis, and stress defense [6]. Their high concentration decreased growth because of nutrient uptake interference, oxidative stress, nutrient imbalance, and cellular toxicity [39]. The optimal concentrations of FeO NPs (50 ppm) and MnO/Cu NPs (75 ppm) were determined based on growth parameters, photosynthetic pigments, and oxidative stress. A significant increase in plant biomass and chlorophyll content was observed at these concentrations, while higher concentrations induced oxidative stress (MDA, H_2_O_2_ accumulation) and growth inhibition. Phytotoxicity thresholds were observed by comparing plant response across different treatments, ensuring that the selected doses provided maximum growth without toxicity. Overall, compared with MnO and Cu NPs, FeO NPs have a lower optimal threshold, highlighting their differential impact on maize growth. A similar trend was observed for the photosynthetic contents. However, above 50 ppm, FeO NPs induced oxidative stress, damaging chloroplast structures and reducing photosynthesis pigment levels, suggesting a lower toxicity threshold. MnO NPs showed the highest chlorophyll content because manganese is essential for water-splitting in photosystem II and plays a significant role in chlorophyll synthesis and stability. Higher concentrations, however, may have caused oxidative stress or metal toxicity. Similarly, Cu NPs improved chlorophyll content, as they may act as electron transporters in photosynthesis [19]. However, excess copper can interfere with cellular homeostasis and trigger oxidative stress, limiting further accumulation of photosynthesis pigment. These results suggest that FeO NPs have a lower effective threshold (50 ppm) than MnO and Cu NPs (75 ppm), confirming their differential impact on maize. Moreover, the application of FeO NPs increased the SOD and POD activities at 50 ppm, with a decrease observed at higher concentrations. MnO and Cu NPs increased the SOD and POD contents at 75 ppm, but their activities decreased significantly at 100 ppm. FeO NPs increase the enzyme activity, as they may increase the ability of cells to neutralize ROS, and MnO NPs may support the mitochondrial form of SOD. Copper may act as a cofactor in antioxidant enzymes, but higher concentrations appear to produce ROS, which the enzymes could not counteract. In the case of oxidative stress, the minimum H_2_O_2_ concentration was observed at 50 ppm FeO, and 75 ppm MnO and Cu NPs. This reduction likely occurs because metal oxide nanoparticles may increase the antioxidant enzyme activities (e.g., catalase, superoxide dismutase) that help to neutralize H_2_O_2_ [40,41]. However, at higher concentrations, H_2_O_2_ levels increased, indicating a shift to pro-oxidant effects. The likely reason is that excessive NP presence may disrupt cellular processes, causing an overproduction of reactive oxygen species (ROS) and overwhelming the cells’ antioxidant defenses, leading to oxidative stress [42]. Figure 11 shows that nanoparticles boost plant stress resistance through the activation of the antioxidant mechanism. Stressed plants produce reactive oxygen species (ROS), which cause harmful oxidative damage to their structures. The application of NPs through foliage activates antioxidant enzymes including SOD, CAT, and elements within the ascorbate–glutathione cycle which inhibits ROS production. After this antioxidant-activating process, plants become more resistant to environmental stress through improved photosynthetic efficiency and reduced oxidative stress [43,44]. These findings suggest that a moderate dosage of NPs can be beneficial for reducing oxidative damage and that higher concentrations can induce oxidative stress due to cellular toxicity and redox imbalance, emphasizing the need for controlled NP use to avoid adverse effects. Therefore, a 50 ppm concentration for FeO, and a 75 ppm concentration for MO and Cu NPs were further used to determine their impact on maize crops under drought stress.

Drought stress decreases physical growth parameters such as leaf size, stem height, and overall biomass by disrupting water uptake, nutrient absorption, and metabolic functions essential for plant growth. The results of this study revealed that the foliar application of iron oxide nanoparticles (FeO NPs), manganese oxide nanoparticles (MnO NPs), and copper nanoparticles (Cu NPs) significantly enhanced the physical growth parameters (SL, RL, FSW, and FRW) of maize under different levels of drought stress. The results showed that the application of all nanoparticles significantly increased the growth parameters at different levels of drought stress. The greatest increase in growth parameters was observed with the application of FeO NPs, compared with MnO and Cu nanoparticles. Nanomaterials positively affect the physical growth parameters of maize by increasing nutrient uptake, improving water retention, and stimulating growth-promoting hormones. They may increase the availability of essential nutrients like nitrogen, phosphorus, and potassium by acting as carriers, leading to improved root and shoot growth [42]. Additionally, they increase photosynthetic efficiency and stress tolerance, resulting in larger leaves, taller stems, and higher biomass accumulation. Ahmad et al. [45] studied the application of zinc oxide nanoparticles and reported that their application improved the growth and yield of maize by increasing nutrient availability and uptake efficiency.

Furthermore, drought stress downregulates chlorophyll content by degrading chloroplasts. However, the application of nanoparticles (FeO NPs, MnO NPs, and Cu NPs) significantly improved the chlorophyll and carotenoid contents of maize in comparison to control. The increase in chlorophyll content might be due to increasing enzymatic activity. These nanoparticles might help to mitigate drought stress and increase total chlorophyll content and carotenoids in crop plants by increasing nutrient uptake and promoting enzymatic activities critical for pigment biosynthesis [46]. According to Wahab et al. [42], iron oxide nanoparticles improve soil water-holding capacity, allowing plants to access water more efficiently during drought conditions, thereby maintaining cellular turgor and supporting sustained growth. Meanwhile, Nair et al. [47] reported that zinc oxide (ZnO) nanoparticles increase the availability of zinc, an essential micronutrient for chlorophyll and carotenoid content, by activating enzymes involved in their biosynthesis pathways. NPs may also stimulate the production of stress-related proteins and antioxidants, protecting chlorophyll from oxidative damage and maintaining high levels of chlorophyll and carotenoids under stress conditions [48]. These mechanisms collectively increase photosynthetic efficiency and overall plant health, leading to improved crop yields.

However, the foliar application of FeO, MnO, and Cu NPs increased the levels of antioxidants (APX, CAT, SOD, and POD) in maize under drought stress to different extents. The maximum antioxidative activity was observed for FeO NPs, followed by MnO and Cu NPs, respectively. The increase in antioxidant activity might be attributed to the crucial role of iron in various enzymatic processes, including those involved in the antioxidative defense system. This increase in antioxidant activity indicated a strengthened antioxidative defense mechanism crucial for maintaining cellular homeostasis under stress conditions [49]. MnO nanoparticles also play a vital role in the antioxidative defense system, particularly in the functioning of enzymes such as antioxidants. Cu nanoparticles are involved in various physiological processes, including the antioxidative defense system [50]. However, excessive copper might lead to toxicity, which may explain the less pronounced effect than that of FeO and MnO nanoparticles. The oxidative stress attributes were also measured, and it was observed that FeO, MnO, and Cu NPs significantly reduced MDA and H_2_O_2_ content under drought stress. These results indicated that metallic nanoparticles decreased MDA and H_2_O_2_ content in plants. The availability of iron supports the production of antioxidant enzymes, which reduce lipid peroxidation and lower MDA and H_2_O_2_ levels [51]. However, an adequate manganese supply through MnO nanoparticles enhances the antioxidative capacity of plants, improving their resilience to oxidative stress. The nanoparticles may increase the activity of antioxidant enzymes, which helps scavenge the reactive oxygen species (ROS) and reduce lipid peroxidation. Similarly, a study by Khan et al. [52] reported that silver (Ag) nanoparticles decreased MDA and H_2_O_2_ content in drought-stressed maize crops by improving their antioxidative response and reducing oxidative damage. A study by El-Saadony et al. [53] found that zinc oxide (ZnO) nanoparticles significantly reduced MDA and H_2_O_2_ levels in maize under drought conditions. Optimal concentrations of nanoparticles enhance antioxidant enzyme activity by providing essential metal cofactors, increasing ROS scavenging, and maintaining cellular balance. Excessive concentrations, however, can cause toxicity, disrupt enzyme function, and induce oxidative stress [26]. Irshad et al. [54] studied chemical and green synthesis of titanium dioxide nanoparticles on cadmium (Cd) uptake in wheat plants. Leaf extracts of two plant species, *Trianthema portulacastrum* (horse purslane), *Chenopodium quinoa* (quinoa) were used for green synthesis while the sol–gel method was used for the chemical preparation of TiO_2_ NPs. Results showed that TiO_2_ NPs significantly enhanced the plant height, length of spikes photosynthesis, and antioxidant attributes compared to chemical synthesis.

FeO, MnO, and Cu nanoparticles’ applications in agriculture have both environmental advantages and safety challenges. Unlike chemically synthesized nanoparticles, biosynthesized NPs use plant extract as a reducing agent, minimizing the release of hazardous by-products and reducing chemical waste accumulation. However, the long-term environmental impact of nanoparticle accumulation in soil and water systems remains a concern. Excessive NP exposure may alter soil microbial communities, affect nutrient cycling, and pose potential risks to non-target organisms [55]. Scaling up laboratory findings for field applications involves optimizing nanoparticle synthesis for large-scale production while maintaining stability and bioactivity. Field trials are essential to validate effectiveness and assess environmental safety, including nanoparticle interactions with soil and plants. Economic feasibility and regulatory compliance must be evaluated to ensure commercial adoption in agriculture. To ensure safe application, dose optimization, and controlled release, formulations should be prioritized. Regulatory frameworks must address nanotoxicity, bioavailability, and persistence in agricultural ecosystems. This research not only confirms the positive impacts of nanoparticle application on maize under drought conditions but also highlights the optimization of nanoparticles to ensure environmental safety and agricultural sustainability.

The biosynthesis of FeO, MnO, and Cu nanoparticles is a cost-effective and sustainable alternative compared to conventional chemical synthesis. The use of plant extract for the biosynthesis of nanoparticles is energy efficient, requires low temperatures for reduction, and generates less toxic waste. A cost-benefit analysis showed that biosynthesized NPs can be produced at 25–30 dollars per kg, compared to 300–400 dollars per kg for chemically synthesized NPs. Meanwhile, field trials showed that biosynthesized NPs increase maize yield by 26–30% under drought stress, providing a higher return on investment (3.5:1 ratio) than traditional approaches. Biosynthesis reduces costs and environmental impact; large-scale application requires standardization and regulatory approval [56]. Overall, the economic analysis supports biosynthesized NPs’ eco-friendly solutions for improving crop productivity under drought stress. Future studies could further refine the application techniques and concentrations of nanoparticles to maximize their efficacy and minimize any potential environmental risks.

## 4. Materials and Methods

### 4.1. Biosynthesis of Nanoparticles

Fresh leaves of *Conocarpus erectus* L. were collected and washed with distilled water to remove dust particles. The washed leaves were dried in sunlight and then ground into a fine powder. A total of 5 g of powdered leaves was added to 50 mL of distilled water and heated at 75 °C for 20 min. This temperature and duration were chosen to ensure efficient extraction of bioactive compounds while preventing their degradation. The resulting solution was filtered via Whatman filter paper, and stored at 4 °C in a glass jar for further use. FeSO_4_·7H_2_O (the Sigma-Al CAS number: 7782-63-0), MnCl_2_·4H_2_O (the Sigma-Al CAS number: 13446-34-9), and CuSO_4_·5H_2_O (the Sigma-Al CAS number: 7758-99-8) were utilized for the synthesis of the FeO, MnO, and Cu NPs. The reaction was conducted at a controlled temperature of 60 °C and was maintained on a hot plate. To optimize nanoparticle formation, the pH of the reaction mixture was continuously monitored via a digital pH meter and adjusted to pH 7.5 by the gradual addition of NaOH and HCl as needed. The reaction mixture was stirred at a constant speed of 500 rpm for 2 h, followed by sonication at 60 °C for 1 h to increase nanoparticle stability and dispersion. The nanoparticles were oven-dried and ground into fine powder. The powder of the nanoparticles was then subjected to a muffle furnace for calcination at 600 °C for approximately 5 h, and stored for characterization and further applications. The yield of the nanoparticles was calculated by dividing the dry weight of the synthesized nanoparticles by the initial weight of the precursor salt and then multiplying by 100 to express it as a percentage.

### 4.2. Characterization of Nanoparticles

The suspensions of nanomaterials were initially subjected to UV-Vis spectroscopy to confirm the synthesis of the particles. Moreover, the shape of the biosynthesized nanoparticles was examined by scanning electron microscopy SEM (SEM LEO 1530, Germany) following the protocol of Firisa et al. [57]. The nanomaterials were coated with aluminum to increase their conductivity and placed in the SEM chamber. The high-resolution images were obtained by adjusting focus, brightness, and contrast. X-ray diffraction analysis was used to determine the crystallinity and size of synthesized nanomaterials. This analysis was carried out via a thermoscientific diffractometer (PANalytical X’PERT PRO, USA) following the method described by Jassim et al. [58], while the size was calculated via Debye Scherrer’s formula (D = Kλ/β cosθ). The attached functional groups to the surface of particles were examined using Fourier transform infrared spectroscopy (FTIR-Bruker TENSOR-27, Germany) following the protocol described in Batool et al. [30].

### 4.3. Optimization of Nanomaterials

After characterization, a pot experiment was conducted to evaluate the threshold level of the particles. Maize seeds (Cimmyt-Pak) were collected from the Ayub Agricultural Research Institute, Faisalabad, Pakistan. Clay loam soil was collected from the agricultural side and passed through a sieve (2 mm) for homogenization. The experiment was conducted under controlled conditions to ensure optimal plant growth. The soil used in pots had a conductivity of 2.7 dS m^−1^ and a pH of 7.8. Nutrient analysis revealed that soil contained 0.65% nitrogen, 11.9 ppm phosphorus, 159 ppm potassium, and 0.76% organic matter. Environmental factors such as temperature, humidity, light intensity, and soil moisture were regularly monitored to maintain consistency throughout the experimental period. A total of 1 kg of soil was added to fifteen pots to optimize the dosage of the nanoparticles. Approximately 5 seeds were sown in each pot and thinned to 3 after 1 week of germination. Different concentrations (0 ppm, 25 ppm, 50 ppm, 75 ppm, and 100 ppm) of each nanomaterial were applied after 7 days of germination. The selected concentration ranges (0–100 ppm) were selected on the basis of the findings of Usman et al. [26], who determined the threshold levels of these nanoparticles. The seedlings were harvested after 21 days. Various morphological (shoot length, root length, shoot weight, and root weight), photosynthetic (chlorophyll a, b, total chl, and carotenoids), antioxidants (superoxide dismutasenand peroxidase), and oxidative stress (hydrogen peroxide) parameters were evaluated after the maize crop was harvested. The optimized dosages of FeO, MnO, and Cu NPs were further used for the second experiment. All the parameters were repeated in the second experiment using the optimized nanoparticle dose.

### 4.4. Experimental Conditions

The pot-based study was conducted at the Environmental Microbiology and Biotechnology Laboratory, Department of Environmental Sciences, GCUF, Faisalabad, Pakistan (31.4° N, 73.06° E). The greenhouse was subjected to the following environmental parameters: a temperature of approximately 30 °C, a moisture content of 44–96%, and 13–15 h of daylight. The Clay Loam soil was again added to the pot with a capacity of 5 kg. The experiment was completely randomized in design with three replicates of each treatment. The total number of pots was 36. About 10 seeds were sown in each pot and thinned to 3, after two weeks of germination. This experiment was conducted in August. The optimized dosage of each nanoparticle was applied under different levels of drought stress (50% FC, 75% FC, and 100% FC). The crop was harvested after 45 days of germination. The following parameters were evaluated after harvesting.

### 4.5. Morphological Parameters

The shoot length (SL), root length (RL), shoot weight (SW), and root weight (RW) were measured using a measuring balance scale.

### 4.6. Photosynthetic Parameters

The contents of carotenoids, total chlorophyll, and chlorophyll a and b were determined after harvesting maize via the protocol described by Palta [59]. About 0.5 g of fresh leaves was ground in 80% acetone. The samples were kept at −4 °C for the entire night. The resulting mixture was then centrifuged for ten minutes at 10,000 rpm. The photosynthetic content was estimated using a spectrophotometer at 633 nm, 645 nm, and 480 nm, and calculated using the following formula,

T. Chl. = [20.2(OD_645_) − 8.02(OD_663_)] × v/w × 1/1000

Chl. a = [12.7(OD_663_) − 2.69(OD_645_)] × v/1000 × w

Chl. b = [22.9(OD_645_) − 4.68(OD_663_)] × v/1000 × w

A Car. µg/g FW = OD_480_ + (0.114 × OD_663_) × (0.638 × OD_645_)

where Car = A Car/Em 100% × 100, emission = Em 100% = 2500, OD = absorbance at corresponding wavelength, V = volume of the extract (mL), and W = weight of the fresh leaf tissue (g).

### 4.7. Antioxidant Estimation

The antioxidant enzyme activities were assessed following standardized extraction protocols. Leaf samples (0.5 g) were homogenized in 50 mM phosphate buffer (pH 7.8) containing 1 mM EDTA and 0.1% polyvinylpyrrolidone (PVP) at 4 °C. The homogenate was centrifuged at 12,000 rpm for 15 min at 4 °C and the supernatant was collected for enzymatic assays. The SOD, POD, and CAT activities were calculated via the protocol developed by Zhang [60]. Moreover, the protocol of Dhindsa et al. [61] was utilized to evaluate the APX activity. The absorbance was measured at specific wavelengths (SOD: 560 nm, POD: 470 nm, CAT: 240 nm, APX: 290 nm) using the spectrophotometer. Enzyme activities are expressed in units per milligram of protein and were calculated using extinction coefficients.

### 4.8. Oxidative Stress Estimation

MDA was evaluated following the method of Zhang and Kirkham [62]. For the estimation of MDA, 0.5 g of maize leaves were ground in 0.1% of 5 mL trichloroacetic acid (TCA). A total of 0.5 mL of the enzyme extract from the homogenized material was mixed with 2 mL of 0.5% thiobarbituric acid (TCA), which was made with 20% TCA. The mixture was heated to 95 °C for 50 min and then allowed to cool to room temperature. The combination was then centrifuged for 10 min at 10,000 rpm, and the absorbance of each sample was measured using the spectrophotometer at 600 and 532 nm.

H_2_O_2_ was calculated following the method described by Dhindsa et al. [61]. For the estimation of H_2_O_2,_ 0.1 g of fresh maize leaves was added to 5 mL of 0.1% (*w*/*v*) trichloroacetic acid in an ice tub to prevent oxidation. The mixture was subsequently centrifuged, after the addition of 1 mL of potassium iodide, 0.5 mL of potassium phosphate, and 0.5 mL of enzyme extract supernatant. The resulting material was allowed to cool at room temperature for 10 min. The absorbance was measured at 390 nm using a spectrophotometer.

### 4.9. Statistical Analysis

In statistical analysis for pairwise comparisons, post hoc analysis was conducted using Fisher’s least significant difference (LSD) test at *p* ≤ 0.05 to determine statistically significant differences among treatments. Analysis of variance (ANOVA) was performed using Statistix 8.1 software, and results were presented as mean ± standard deviation (SD) based on three biological replicates (n = 3).

## 5. Conclusions

The present study demonstrated that biosynthesized iron oxide nanoparticles (FeO NPs), manganese oxide nanoparticles (MnO NPs), and copper nanoparticles (Cu NPs) significantly mitigate drought stress in maize (*Zea mays* L.). The optimization of nanoparticle concentrations and their subsequent application under varying drought conditions positively influences growth parameters (shoot and root length, shoot and root weight), as well as physiological and biochemical parameters (chlorophyll content, oxidative stress markers, and antioxidants). Notably, the foliar application of iron oxide nanoparticles resulted in the most significant improvement in plant growth and physiological and biochemical parameters under drought stress, followed by the application of manganese oxide nanoparticles and copper nanoparticles. These findings indicate that different concentrations of FeO NPs, MnO NPs, and Cu NPs effectively increase maize growth and resilience under drought conditions, providing a potential strategy for improving crop yield and nutritional value in water-limited environments.

## Data Availability

Data are contained within the article.
